# Pediatric Severe Sepsis Prediction Using Machine Learning

**DOI:** 10.3389/fped.2019.00413

**Published:** 2019-10-11

**Authors:** Sidney Le, Jana Hoffman, Christopher Barton, Julie C. Fitzgerald, Angier Allen, Emily Pellegrini, Jacob Calvert, Ritankar Das

**Affiliations:** ^1^Dascena Inc., Oakland, CA, United States; ^2^Department of Emergency Medicine, University of California, San Francisco, San Francisco, CA, United States; ^3^Department of Anesthesiology and Critical Care Medicine, Children's Hospital of Philadelphia, Philadelphia, PA, United States; ^4^Department of Anesthesiology, Perelman School of Medicine, University of Pennsylvania, Philadelphia, PA, United States

**Keywords:** pediatric severe sepsis, prediction, machine learning, electronic health records, early detection

## Abstract

**Background:** Early detection of pediatric severe sepsis is necessary in order to optimize effective treatment, and new methods are needed to facilitate this early detection.

**Objective:** Can a machine-learning based prediction algorithm using electronic healthcare record (EHR) data predict severe sepsis onset in pediatric populations?

**Methods:** EHR data were collected from a retrospective set of de-identified pediatric inpatient and emergency encounters for patients between 2–17 years of age, drawn from the University of California San Francisco (UCSF) Medical Center, with encounter dates between June 2011 and March 2016.

**Results:** Pediatric patients (*n* = 9,486) were identified and 101 (1.06%) were labeled with severe sepsis following the pediatric severe sepsis definition of Goldstein et al. (1). In 4-fold cross-validation evaluations, the machine learning algorithm achieved an AUROC of 0.916 for discrimination between severe sepsis and control pediatric patients at the time of onset and AUROC of 0.718 at 4 h before onset. The prediction algorithm significantly outperformed the Pediatric Logistic Organ Dysfunction score (PELOD-2) (*p* < 0.05) and pediatric Systemic Inflammatory Response Syndrome (SIRS) (*p* < 0.05) in the prediction of severe sepsis 4 h before onset using cross-validation and pairwise *t*-tests.

**Conclusion:** This machine learning algorithm has the potential to deliver high-performance severe sepsis detection and prediction through automated monitoring of EHR data for pediatric inpatients, which may enable earlier sepsis recognition and treatment initiation.

## Introduction

Sepsis is a high-impact condition that affects both adults and children. In 2001, the total burden of sepsis-spectrum syndromes in the United States was estimated at $16.7 billion and 215,000 deaths annually (2). In 2007, the mean, per-hospitalization cost of severe sepsis was estimated to be $47,126 (3), and a recent study assessed that sepsis is responsible for as many as 5.3 million deaths per year globally (4). Pediatric sepsis in particular causes over 6,500 deaths annually in the United States, with an estimated $4.8 billion burden of care, at approximately $64,280 per hospitalization (5). Moreover, survivors can suffer both short-term (6) and long-lasting impacts (7). Relative to that of adult sepsis, the literature of pediatric sepsis is less developed (8). This includes consensus definitions of pediatric sepsis (1, 9, 10), which may not match clinicians' diagnoses in practice (11).

As is true for adult sepsis (12, 13), many studies show that early and aggressive treatment of pediatric sepsis with antibiotics and fluid resuscitation correlates with better outcomes (14–19). Traditionally, generalized disease severity scoring systems have been used for sepsis detection; however, these lack specificity for pediatric sepsis. For example, while the Systemic Inflammatory Response Syndrome (SIRS) criteria have been adapted for pediatric patients and incorporated into current pediatric sepsis definitions (1), they are intended to assess inflammatory responses from both infection and other causes of systemic inflammation, resulting in criteria that are sensitive but not specific for sepsis. In some cases, scoring systems for non-specific pediatric disease severity or mortality are applied to the task of recognizing pediatric sepsis, such as the Pediatric Logistic Organ Dysfunction score (PELOD-2) (20, 21). PELOD-2 is a continuous scale that allows assessment of the severity of cases of MODS in the PICU and which includes 10 variables (Glasgow Coma Score, pupillary reaction, lactatemia, mean arterial pressure, creatinine, PaO_2_/FiO_2_ ratio, PaCO_2_, ventilation, WBC count, and platelet count), involving five organ dysfunctions (neurologic, cardiovascular, renal, respiratory, and hematologic) (20).

In the absence of specialized pediatric sepsis scores, some hospitals have implemented home-grown computerized sepsis prediction systems, which may benefit from site specificity (22). Computerized prediction systems offer a compelling alternative to manual application of generalized scoring systems. Such systems may access electronic health record (EHR) data for clinical decision support. These systems have the potential to identify septic patients who might otherwise have a delay in diagnosis or be missed entirely, and could provide early warning of sepsis for hospitalized pediatric patients on hospital wards or in intensive care. Studies in adults show that the setting of hospital-acquired sepsis among inpatients is both distinct and substantially more deadly (23–25), and children with hospital-acquired sepsis have higher risk of delayed sepsis care than those presenting to an emergency department (18).

Machine-learning (ML)-based approaches have the potential for increased sensitivity and specificity by training on sepsis patient data (26–28), and can be easily customized using site- or population-specific data, resulting in improved performance relative to generic scoring systems (29–31). While ML-based systems have been applied to prediction of sepsis in neonatal patients, conditioned on the availability of real-time waveform data (32, 33) or extensive sets of laboratory and historical data (34), they have not previously been applied to EHR-based prediction for the older pediatric inpatient population. If successful, such predictors could provide easily-accessible, site-customized early and accurate sepsis warning for pediatric patients. In the experiments discussed below, our objective was to create and demonstrate a customized, high-performance ML-based prediction tool for pediatric severe sepsis.

## Methods

### Data Set

In these experiments, we used de-identified chart data from pediatric (ages 2–17 years) inpatient and emergency encounters at the University of California San Francisco (UCSF) Medical Center, from June 2011 to March 2016, inclusive (35). See Table 1 for additional details on data inputs. Neonates and infants under the age of 2 years have immature adaptive and innate immune responses (36), and require separate analysis beyond the scope of this work. The original UCSF data collection did not impact patient safety, as all data were de-identified in accordance with the Health Insurance Portability and Accountability Act (HIPAA) Privacy Rule prior to commencement of this study and no individual patient data were linked prior to being de-identified. Hence, this study constitutes non-human subjects research which does not require Institutional Review Board approval.

**Table 1:** Predictor variables used in this study.

**Demographics**	**Age**
Vital signs	Heart rate Respiratory rate Peripheral oxygen saturation (SpO_2_) Temperature Systolic blood pressure Diastolic blood pressure
Other clinical variables (not required for inclusion)	Glasgow Coma Scale (GCS) White Blood Cell (WBC) count Platelet count

Encounters were removed if the recorded patient age was <2 or more than 17 years (1). In addition, encounters were removed if they were missing any of the required measurements (patient age, diastolic and systolic blood pressures, heart rate, temperature, respiration rate, and peripheral oxygen saturation) to be used in training and prediction; while supplemental measurements (Glasgow Coma Score, white blood cell count, and platelet count) were passed to the training and testing routines, their presence was not required. From 11,619 encounters with appropriate ages, 9,715 remained after checking for the required measurements. As a final step, encounters with severe sepsis onset too early in the stay (<6 h after the start of the patient record) were removed (see Experimental Procedures). After removing encounters for these onset times, a total of 9,486 encounters remained for training and testing. Of these examples, 101 (1.06%) were determined to have severe sepsis (see Gold Standard).

### Data Processing and Screening

The UCSF EHR data were organized into a SQL database and custom queries were used to extract the vital sign, lab report, and other data used in our experiments. These patient records were prepared by Dascena's proprietary software to provide examples for training and prediction. For encounters meeting the inclusion criteria, observations of vital signs were binned by the hour and simple carry-forward imputation was used when no observation was available for a given hour. Based on these time series data, we constructed a variety of derived features (e.g., approximate Mean Arterial Pressure constructed as a linear combination of systolic and diastolic blood pressures) and calculated the sepsis gold standard for training (see Supplementary Tables 1, 2).

### Gold Standard

The gold standard follows the pediatric severe sepsis definition of Goldstein et al. (1), wherein severe sepsis requires:

- SIRS score of ≥ 2, where at least one of temperature or white blood cell count is abnormal;
- Suspicion of infection, operationalized here as the presence of an International Classification of Diseases (ICD-9) code for septicemia, sepsis, severe sepsis, or septic shock, which might be attached at any time during the encounter (given that the patient meets the SIRS criteria above, this is “sepsis” under the Goldstein criteria); and
- Organ dysfunction.

Under the Goldstein criteria, septic shock is further defined by when the above conditions are met and there is cardiovascular organ dysfunction. Pediatric SIRS criteria, the gold standard, and the organ dysfunction criteria (part of the gold standard) are presented in Supplementary Tables 1–3, respectively. Supplementary Table 4 contains a list of ICD-9 codes used for “suspicion of infection.”

The Goldstein criteria was chosen for use as the gold standard as opposed to Sepsis-3 criteria because Sepsis-3 criteria are based on a patient's Sequential Organ Failure Assessment (SOFA) Score, a severity score which lacks sufficient evidence for use in pediatrics (1, 37, 38). In addition, Sepsis-3 criteria identifies those patients with sepsis and septic shock, which reflect respective mortality rates of 10 and 35% (38). Because mortality rates have been widely reported in the literature to be different in pediatric sepsis as opposed to adult sepsis (5, 36, 39), these rates are not applicable to pediatric populations. Use of Sepsis-3 criteria would preclude direct applicability of our intervention to pediatric patients, including different cognitive, developmental, and disease stages that present in cases of pediatric sepsis as compared to adult sepsis (36).

The gold standard was implemented by electronic chart abstraction, combining data entered into the EHR throughout the encounter with ICD-9 codes. All of these criteria follow Goldstein et al. (1), with modifications necessary for application to the UCSF pediatric data set. These necessary modifications include those allowing for binary white blood cell count (normal vs. abnormal), lack of radiological information, and lack of patient history. Without information on physician intention and examination observations, fluid administration in the presence of low blood pressure was assumed to be an attempt to resuscitate, and the following sections of the Goldstein cardiovascular dysfunction definitions were modified: it was not possible to assign causal attribution for fluid-refractory hypotension, and exam components (core-to-peripheral temperature gap and delayed capillary refill) could not be determined retrospectively. Radiological (bilateral infiltrates) and history components (acute onset and no evidence of left heart failure) were unavailable (see Supplementary Tables for details of implemented criteria).

Sepsis onset was defined as the time when the patient first met the SIRS criteria (if a sepsis-related ICD-9 code is present). Severe sepsis onset was defined as the first time the organ dysfunction criteria was met in a patient who, at some point in their stay, met the SIRS criteria and had a sepsis-related ICD-9 code (is “septic” under our gold standard). Note that this means that our retrospective definition may determine that a patient has “severe sepsis” before they meet the surveillance criteria for being “septic.” For example, if laboratory data fulfilling organ dysfunction definitions was present prior to fever and tachypnea being recorded in vital sign flowsheets, the patient would be labeled with severe sepsis. This feature was deemed necessary both to reflect the clinical process and to avoid difficulties surrounding noisy satisfaction of the thresholds.

### Modeling

All learning conducted in this work was done using boosted ensembles of decision trees (40, 41). Ensemble classifiers combine the output from many “weak” learners, each of which would be insufficient to solve the desired learning problem on its own, creating a strong learner. Each of these weak base learners is a decision tree, constructed by repeatedly and recursively partitioning the feature space, finding thresholds within the features which most optimally decrease entropy, and thus increase information, within the resulting classification groups The appropriate set of branching checks is performed for each tree within the final classifier, traveling along the tree structure until a leaf node (and corresponding risk score) are reached. The risk scores from the individual trees are then aggregated to assign an overall risk score.

Our classifiers were trained on a set of features that included patient age, diastolic and systolic blood pressures, heart rate, temperature, respiration rate, and peripheral oxygen saturation (SpO_2_) (Table 1). As noted above, encounters had to have all of these measurements at some point during their stay to qualify for inclusion in the analyses. Additionally, the values of Glasgow Coma Score, white blood cell count, and platelet count were used if available. The final feature vectors were organized, along with their gold standard labels, into arrays to be passed to the training and prediction routines.

### Experimental Procedures

We compared the performance of the algorithmic sepsis predictor with that of the concurrent, running values of the PELOD-2 and pediatric SIRS scores. These experiments used all patients of at least 2 and ≤ 17 years of age in the data set, treated as one aggregate population. This population was split into four approximately equal-sized “folds” (sets) for 4-fold cross-validation (CV) (41). The CV procedure allows the estimation of generalization performance and its variability, as well as comparison of this performance with the PELOD-2 and SIRS scores, calculated hourly. Due to the original data set's encoding of laboratory values as only normal/abnormal, the affected subscores of PELOD-2 were approximated with 1 point for abnormal and 0 points for normal. We computed a variety of metrics on the performance of the resulting classifiers (and the PELOD-2 and SIRS scores) on the test folds. We determined statistical significance using one-tailed paired *t*-tests, where each pair constituted the AUROC performance of two different classification methods, measured on the same test fold. This paired *t*-test has a sample size of 4, as we are comparing 4 AUROC performances, paired by test set on which they were evaluated The *p*-value threshold for significance was fixed at 0.05 for all comparisons. We repeated these experiments for pre-onset offsets of 0, 1, 2, 3, and 4 h, examining our system's ability to learn pre-onset patterns in septic patients.

## Results

The demographic characteristics, inclusion flowchart, and feature importance of the data set are presented in Table 2, Supplementary Figures 1, 2, respectively. The overall prevalence of severe sepsis in this pediatric population (aged 2–17, inclusive) was 1.06% (47.52% male and 52.48% female). Among patients with severe sepsis, 72.94% were above the age of 5 years.

**Table 2:** Demographic information of pediatric inpatients at UCSF from June 2011 to March 2016, inclusive.

**Characteristic**		**Overall**		**Severe sepsis**	
		**Count**	**Percent (%)**	**Count**	**Percent (%)**
Gender	Female	4,706	49.61	48	47.52
	Male	4,780	50.39	53	52.48
Age	2–5	2,567	27.06	29	28.71
Overall: Median	6–12	3,476	36.64	34	33.66
10, IQR (5–14) Severe sepsis: Median 9, IQR (4–14)	13–17	3,443	36.30	38	37.62
Length of stay	0–2	5,021	52.93	15	14.85
(days)	3–5	2,412	25.43	12	11.88
Overall: Median 2,	6–8	849	8.95	21	20.79
IQR (1–5)	9–11	420	4.43	17	16.83
Severe sepsis: Median 9, IQR (5–16)	12+	784	8.27	36	35.64
In-hospital death	Yes	47	0.50	7	6.93
	No	9,439	99.50	94	93.07

**Figure 1:**
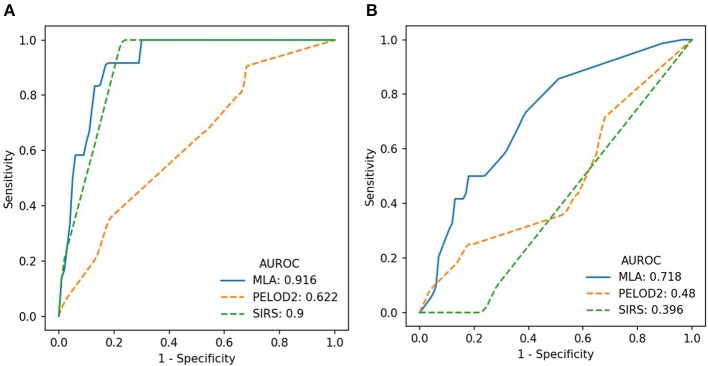
**(A)** ROC curves (averaged across the four test folds) for the machine learning algorithm (MLA), PELOD-2, and SIRS at time of onset. **(B)** ROC curves (averaged across the four test folds) for the MLA, PELOD-2, and SIRS at 4 h pre-onset.

We evaluated predictive performance of the ML-based predictor by training and testing at hourly intervals from sepsis onset and through 4 h before onset. Figures 1A,B show the performance of the algorithm at onset and 4 h before onset in terms of Receiver Operating Characteristic (ROC) curves, which show the tradeoff between sensitivity (the fraction of severe sepsis patients that were classified as severe sepsis) and specificity (the fraction of severe sepsis-negative patients that were classified as severe sepsis). The ML-based predictor's ROC curve is improved over the PELOD-2 and SIRS curves, and has a larger area under the curve (i.e., larger AUROC), which indicates increased accuracy.

**Figure 2:**
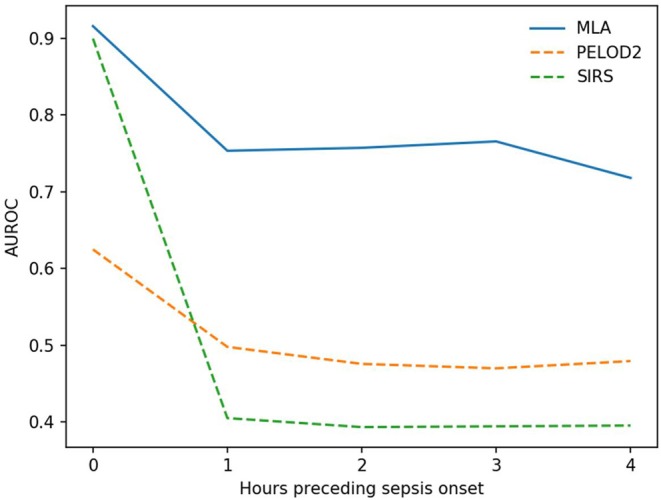
Average AUROC over a prediction horizon. These AUROC differences are statistically significant for the machine learning algorithm (MLA) vs. PELOD-2 at all hours pre-onset (*p* < 0.05) and vs. SIRS at all hours pre-onset (*p* < 0.05) with the exception of the 0 h comparison. This non-significant comparison against SIRS at 0 h pre-onset had a *p*-value of 0.0977.

Figure 2 shows how cross-validation fold-averaged AUROC varies as a function of prediction horizon in hours for each prediction system. These comparisons are statistically significant (*p* < 0.05, one-tailed pairwise *t*-test) for 1 and 4 h pre-onset (PELOD-2) and 0, 1, and 4 h pre-onset (SIRS).

Table 3 presents a set of detailed performance metrics for the algorithm, SIRS, and PELOD-2. Apart from AUROC, these performance metrics are a function of a chosen operating point (i.e., a point on the ROC curve where the sensitivity was the largest possible value ≤ 0.80). The diagnostic odds ratio (DOR), a global measure for comparing diagnostic accuracy between diagnostic tools, is represented here as the ratio of the odds of a true positive prediction of severe sepsis in patients who developed severe sepsis relative to the odds of a false positive prediction of severe sepsis in patients who did not develop severe sepsis. The DOR is highest for the MLA predictor (vs. PELOD-2 and SIRS) at onset and 4 h pre-onset.

**Table 3:** Performance metrics for the machine learning algorithm and pediatric scoring systems.

	**MLA (onset) mean (SE)**	**PELOD-2 (onset) mean (SE)**	**SIRS (onset) mean (SE)**	**MLA (4 h pre-onset)**	**PELOD-2 (4 h pre-onset)**	**SIRS (4 h pre-onset)**
				**mean (SE)**	**mean (SE)**	**mean (SE)**
AUROC	**0.916 ± (0.053)**	0.622±(0.093)	0.900±(0.029)	**0.718 ± (0.182)**	0.482±(0.082)	0.396±(0.051)
Sensitivity	0.750±(0.000)	**0.805 ± (0.078)**	0.775±(0.157)	**0.750 ± (0.167)**	0.707±(0.089)	0.067±(0.141)
Specificity	**0.940 ± (0.049)**	0.383±(0.064)	0.861±(0.067)	0.700 ± (0.180)	0.351±(0.043)	**0.740 ± (0.013)**
DOR	**64.438 ± (70.499)**	3.023±(1.548)	28.112±(30.507)	**8.384 ± (6.104)**	1.454±(0.607)	0.271±(0.573)

*For each metric and each time (onset or 4 h pre-onset), the best result is bolded. This procedure chose an operating point from the ROC curve where the sensitivity was the largest possible value ≤ 0.80; the selected PELOD-2 and SIRS sensitivity values for 4 h pre-onset prediction were considerably below this value, allowing them to obtain favorable tradeoffs in some of the other metrics. MLA is machine learning algorithm. SE is the standard error and DOR is the diagnostic odds ratio*.

## Discussion

These experiments demonstrate that the ML-based sepsis prediction system can predict severe sepsis onset with AUROC performance superior to that of existing pediatric organ dysfunction and inflammatory response scoring systems (Table 2, Figures 1, 2). These comparisons were statistically significant vs. PELOD-2 (organ dysfunction) at 1 and 4 h pre-onset and vs. SIRS at 0, 1, and 4 h pre-onset. This superiority is also visible in other metrics. Early and accurate recognition of pediatric sepsis is essential, particularly in high-risk inpatient populations, as this could lead to earlier treatment initiation and potentially improve patient outcomes. Effective methods to improve pediatric sepsis recognition are lacking and have been called for as part of national pediatric sepsis improvement collaboratives.

This study contributes to the ongoing body of literature on the use of machine learning for the prediction of sepsis (42–44). Komorowski et al. (42) developed the AI Clinician, a reinforcement learning model, and retrospective results indicated that patients who received treatments similar to those the model recommended had the lowest mortality (42). The AI Clinician was developed with a variety of data inputs, some of which have limited EHR availability or would not have been immediately available to the clinician at the time of treatment. It provides useful treatment recommendations when no gold standard for treatment exists, which is a significant current need in the US healthcare delivery landscape. However, while Komorowski et al. derived some insight into the tool's interpretability by estimating relative importance of model parameters, CDS tools that recommend treatment plans must also provide clinicians with transparency along with treatment recommendations, such that clinicians can easily and efficiently interpret the basis for those recommendations (42, 43). Nemati et al. (44) developed the Artificial Intelligence Sepsis Expert algorithm, a sepsis prediction model derived from a combination of electronic medical record (EMR) and high-frequency physiologic data (44). Retrospective results indicated that the model could accurately predict the onset of sepsis in an ICU patient 4–12 h prior to clinical recognition (44). These studies represent important contributions to the field of machine learning and its application to sepsis identification and prediction. However, to the best of our knowledge, none of these existing machine-learning based systems provide early warning for the complex, heterogeneous pediatric sepsis inpatient population, which can present very differently than adult sepsis due to the wide ranges of physiological baseline states (1) and sepsis manifestations in pediatric patients (45), their “tremendous physiological reserve,” (46) which tends to mask early symptoms, and the difficulties posed by numerous comorbidities and treatments (47). Therefore, there remains a significant clinical need for a sensitive and specific EHR-based pediatric sepsis prediction system.

The ML-based system analyzed in this study can be a useful means of continually assessing pediatric patients' likelihood of developing severe sepsis through automatic monitoring of the patient EHR. This clinical problem represents a significant opportunity for clinical decision support, as it is critical to both provide monitoring for this particularly vulnerable population and avoid excessive numbers of false alarms. Our machine learning algorithm outperforms the PELOD-2 and pediatric SIRS scoring systems, indicating that it has the potential to deliver these essential improvements. While neither PELOD-2 or SIRS are primarily intended for sepsis prediction, they provide a practical baseline for this retrospective study. Further, the UCSF dataset used in this study is a collection of encounters from many hospital wards with varying measurement frequencies and data types, and information is compiled with unique patient identifiers, meaning that measurement types remain consistent across patient encounters. Measurement of vital signs therefore followed similar approaches among the cohort of patients analyzed in this study, which strengthens the generalizability of experimental results.

The gold standard is a possible limitation in the present analysis. First, chart review would provide a superior gold standard, but it is not practical at scale, requiring the present use of our surveillance-type gold standard. Second, by limiting “suspicion of infection” to those who have ICD-9 codes for sepsis-spectrum syndromes, we prevent the gold standard from positively labeling encounters not acknowledged as being on this spectrum; this could mean that this gold standard is under-reporting sepsis prevalence. Further, Weiss et al. (11) compared the clinical diagnoses of severe sepsis by attending physicians with the result of the application of the Goldstein consensus definitions and found that the agreement between the two was only moderate (Cohen's χ, 0.57 ± 0.02, mean ± SE). The current analysis uses only ICD-9 codes and does not use ICD-10 codes, while the study period includes the roll-out of the ICD-10-CM coding system on October 1, 2015 (48). However, ICD-9 codes for sepsis appear with a similar frequency both before and after the roll-out date. Finally, while the gold standard provides a particular onset time based on vital signs and laboratory data, it is difficult to assess how this relates to when severe sepsis onset would be recognized by an attending clinician. There could be additional delays to clinician recognition of the change in physiology and further delays to necessary interventions.

The characteristics of the UCSF pediatric inpatient population may limit generalizability; these data are from a tertiary care center with a heavy representation of organ transplant patients. This population also has a low prevalence of hospital-acquired severe sepsis (<1%), limiting the power of the statistical analyses. Although pediatric severe sepsis in children's hospital PICUs has occurred with increasing prevalence and with increasing associated comorbidities, resource burden, and mortality in recent years (49), generalizability may be limited when applying the methodology used in this study to “sepsis” diagnostic criteria instead of to “severe sepsis” criteria. Despite these limitations, these data provide proof-of-principle that machine learning can identify and predict pediatric sepsis, and could be adapted with increased performance at each center to have better site specificity for each hospital's particular patient population.

Because of the retrospective nature of this work, the dataset was not initially collected with the purpose of machine learning on a cohort of children at risk for sepsis. For future, more complex work in pediatric sepsis prediction via machine learning, an important requirement is obtaining diverse and large data sets. It should be noted that, while pediatric sepsis is generally rare, even secondary-care contexts could benefit from being able to accurately identify early the few cases that do appear, particularly if such capabilities could be integrated into a larger data collection and prediction system, as they can with this predictive algorithm.

In summary, the ML-based sepsis prediction system examined in these experiments outperforms traditional, tabular scoring systems and demonstrates superior performance in predicting pediatric severe sepsis onset. The improved ROC performance offers clinicians and hospitals a variety of useful operating points to suit their sepsis alerting needs. The ROC performance also offers the promise of using the numerical score produced by this algorithm for severe sepsis risk stratification. Using these tools, clinicians will be better able to allocate finite clinical resources, identify pediatric patients before their condition deteriorates, and avoid adverse outcomes.

## Data Availability Statement

The data that support the findings of this study are available from the University of California, San Francisco Medical Center in San Francisco, CA (UCSF), but restrictions apply to the availability of these data, which were used under license for the current study, and so are not publicly available. Data are however available upon reasonable request and with permission of UCSF at (at: https://myresearch.ucsf.edu/research-data-browser-de-identified-export-and-flowsheet-files).

## Ethics Statement

The original UCSF data collection did not impact patient safety and all data were de-identified in accordance with the Health Insurance Portability and Accountability Act (HIPAA) Privacy Rule prior to commencement of this study. Hence, this study constitutes non-human subjects research which does not require Institutional Review Board approval.

## Author Contributions

SL revised and performed the MLA experiments, analyzed and interpreted data, reviewed and revised the manuscript, and approved the final manuscript as submitted. RD conceptualized and designed the study, and approved the final manuscript as submitted. JC, SL, and AA analyzed and interpreted data, reviewed and revised the manuscript, and approved the final manuscript as submitted. JH, JF, EP, and CB reviewed and revised the manuscript, and approved the final manuscript as submitted.

### Conflict of Interest

JH, CB, JC, SL, AA, EP, and RD are employed by Dascena Inc. JF reports receiving research grant funding from Dascena Inc.

## Abbreviations

AUROC: area under the receiver operating characteristic
CV: cross-validation
DOR: diagnostic odds ratio
HER: electronic health record
ICD-9, international classification of diseases: 9th revision
IQR: interquartile range
ML: machine learning
MLA: machine learning algorithm
PELOD: pediatric logistic organ dysfunction score
ROC: receiver operating characteristic
SE: standard error
SIRS: systemic inflammatory response syndrome
UCSF: University of California San Francisco.
