# Supplementary material for: Pediatric Severe Sepsis Prediction Using Machine Learning
Source: Front Pediatr. 2019 Oct 11;7:413. doi: 10.3389/fped.2019.00413 (PMC6798083; doi:10.3389/fped.2019.00413)
Supplement: Supplementary file 1 [file Data_Sheet_1.docx]

Supplementary Material

# Supplementary Figures and Tables


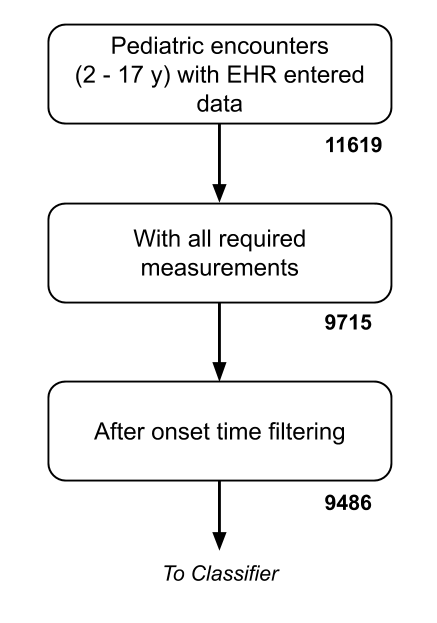


**Supplemental Figure 1**: Inclusion flowchart. The final data set used in training and testing constitutes 9,486 examples, of which 101 (1.06%) are labeled as severely septic.


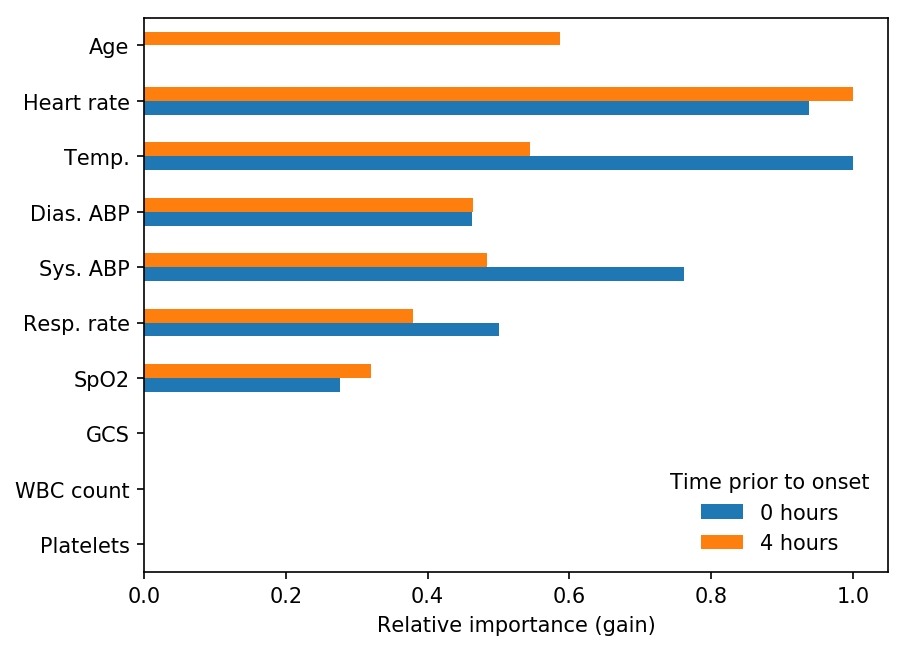

**Supplemental Figure 2:** Feature importance plot. The importance scores are measures of relative information gain (or equivalently, entropy decrease) and are measured during the training of models and averaged across folds. Values presented in this plot are scaled to the maximum importance score for a given prediction time to enable comparison between prediction times.

**Supplemental Table 1:** Pediatric SIRS (modified from Goldstein et al., 2005), for patient age ranges examined in this study. The SIRS score is the total of the number of the four measurements below that are out-of-range. In this data set, white counts are reported only as “normal” or “abnormal.”

|  | Temperature | | Heart Rate | Respiration Rate | White Count |
| --- | --- | --- | --- | --- | --- |
| Patient age (y) | (C, low) | (C, high) | Tachycardia (beats/min) | Tachypnea (breath/min) |  |
| 2-5 | < 36 | > 38.5 | > 140 | > 22 | abnormal |
| 6-12 |  |  | > 130 | > 18 |  |
| 13-17 |  |  | > 110 | > 14 |  |

**Supplemental Table 2:** Pediatric Sepsis, Severe Sepsis, and Septic Shock Gold Standard (after Goldstein et al., 2005).

| Sepsis | Any ICD-9 code for septicemia, sepsis, severe sepsis, or septic shock, plus a SIRS score of ≥ 2, where at least one of temperature or WBC is abnormal. | |
| --- | --- | --- |
| Severe sepsis | Sepsis, plus: | Cardiovascular dysfunction, acute respiratory distress syndrome (ARDS), or ≥ 2 other organ dysfunctions (respiratory, neurologic, hematologic, renal, hepatic) |
| Septic Shock | Severe sepsis, plus: | Cardiovascular organ dysfunction |

**Supplemental Table 3:** Organ dysfunction criteria (modified from Goldstein et al., 2005). *: Substantial modifications required for chart-based implementation. Causal attribution is impossible for fluid-refractory hypotension, and exam components (core-to-peripheral temperature gap and delayed capillary refill) cannot be checked retrospectively. **: Radiological (bilateral infiltrates) and history components (acute onset and no evidence of left heart failure) unavailable. ***: Substantial modifications required for chart-based implementation. Proven need, causal attribution of FiO_2_ and SpO_2_ relationship, and non-elective nature of mechanical ventilation cannot be assessed retrospectively.

| Cardiovascular* | Delivery of 40 ml/kg/hr of IV fluids during an hour-long period, any of: concurrent Systolic BP < 74, 83, 90 mmHg (for patients 2-5, 6-12, or 13-17); IV dopamine of ≥ 5 μg/kg/min; any IV Dobutamine, Epinephrine, or Norepinephrine. |
| --- | --- |
| ARDS** | PaO2/FiO_2_ < 200 mmHg |
| Respiratory*** | PaO2/FiO_2_ < 300 mmHg, PaCO_2_ > 65 mmHg, FiO_2_ ≥ 0.50 |
| Neurologic | Glasgow Coma Scale ≤ 11 |
| Hematologic | Platelets “abnormal” or INR “abnormal” |
| Renal | Serum creatinine “abnormal” |
| Hepatic | Alanine transaminase “abnormal” or total bilirubin “abnormal” |

**Supplementary Table 4**. ICD-9 codes used for “suspected infection” classification.

| ICD-9 code | Name |
| --- | --- |
| 003.1 | Salmonella septicemia |
| 038.0 | Streptococcal septicemia |
| 038.10 | Staphylococcal septicemia, unspecified(038.10) |
| 038.11 | Methicillin susceptible Staphylococcus aureus septicemia |
| 038.12 | Methicillin resistant Staphylococcus aureus septicemia |
| 038.19 | Other staphylococcal septicemia |
| 038.2 | Pneumococcal septicemia |
| 038.3 | Septicemia due to anaerobes |
| 038.40 | Septicemia due to gram-negative organism, unspecified(038.40) |
| 038.41 | Septicemia due to hemophilus influenzae |
| 038.42 | Septicemia due to escherichia coli |
| 038.43 | Septicemia due to pseudomonas |
| 038.44 | Septicemia due to serratia |
| 038.49 | Other septicemia due to gram-negative organisms |
| 038.8 | Other specified septicemias |
| 038.9 | Unspecified septicemia |
| 054.5 | Herpetic septicemia |
| 415.12 | Septic pulmonary embolism |
| 422.92 | Septic myocarditis |
| 449 | Septic arterial embolism |
| 670.20 | Puerperal sepsis, unspecified as to episode of care or not applicable |
| 670.22 | Puerperal sepsis, delivered, with mention of postpartum complication(670.22) |
| 670.24 | Puerperal sepsis, postpartum condition or complication(670.24) |
| 670.34 | Puerperal septic thrombophlebitis, postpartum condition or complication(670.34) |
| 673.31 | Obstetrical pyemic and septic embolism, delivered, with or without mention of antepartum condition(673.31) |
| 771.81 | Septicemia of newborn |
| 785.52 | Septic shock |
| 995.90 | Systemic inflammatory response syndrome, unspecified(995.90) |
| 995.91 | Sepsis |
| 995.92 | Severe sepsis |
| 995.93 | Systemic inflammatory response syndrome due to noninfectious process without acute organ dysfunction |
| 995.94 | Systemic inflammatory response syndrome due to noninfectious process with acute organ dysfunction |
| 998.02 | Postoperative shock, septic(998.02) |
